# Eye-spots in Lepidoptera attract attention in humans

**DOI:** 10.1098/rsos.150155

**Published:** 2015-06-17

**Authors:** Jessica L. Yorzinski, Michael L. Platt, Geoffrey K. Adams

**Affiliations:** 1 Duke Institute for Brain Sciences and Center for Cognitive Neuroscience, Duke University, Durham, NC 27710, USA; 2 Department of Neurobiology, Duke University Medical Center, Durham, NC 27710, USA

**Keywords:** attention, humans, eye-spots, eye-tracking, prey selection

## Abstract

Many prey species exhibit defensive traits to decrease their chances of predation. Conspicuous eye-spots, concentric rings of contrasting colours, are one type of defensive trait that some species exhibit to deter predators. We examined the function of eye-spots in Lepidoptera to determine whether they are effective at deterring predators because they resemble eyes (‘eye mimicry hypothesis’) or are highly salient (‘conspicuous signal hypothesis’). We recorded the gaze behaviour of men and women as they viewed natural images of butterflies and moths as well as images in which the eye-spots of these insects were modified. The eye-spots were modified by removing them, scrambling their colours, or replacing them with elliptical or triangular shapes that had either dark or light centres. Participants were generally more likely to look at, spend more time looking at and be faster to first fixate the eye-spots of butterflies and moths that were natural compared with ones that were modified, including the elliptical eye-spots with dark centres that most resembled eyes as well as the scrambled eye-spots that had the same contrast as the natural eye-spots. Participants were most likely to look at eye-spots that were numerous, had a large surface area and were located close to the insects' heads. Participants' pupils were larger when viewing eye-spots compared with the rest of the insects' body, suggesting a greater arousal when viewing eye-spots. Our results provide some support for the conspicuous signal hypothesis (and minimal support for the eye mimicry hypothesis) and suggest that eye-spots may be effective at deterring predators because they are highly conspicuous signals that draw attention.

## 1. Introduction

The coevolution of predators and prey can lead to dramatic changes in phenotypic traits [1,2]. Selection favours predators that efficiently capture prey and prey that successfully avoid predators. These opposing forces can lead to an arms race in which traits of predators and prey change to counteract decreased fitness levels [3]. However, costs may limit the evolution of these modified traits [4]. Many prey species currently exhibit defensive traits that reduce their chances of predation [5].

Morphological adaptations are one defensive trait that prey use to minimize predation risk [5]. Prey often possess specific traits, like quills or spines, which inflict injury upon potential predators [6]. Their skin can be so thick that it is difficult for predators to puncture [7] and it can be thicker in especially vulnerable areas of the body [8]. Overall body size influences predation rates as well since predators may be unable to consume relatively large prey [9]. Prey coloration can also reduce predation. Some animals have cryptic coloration that closely matches the environments in which they live, making detection by predators less likely [10]. Alternatively, animals may exhibit bright coloration to advertise their unpalatability [11]. Some species, such as butterflies, moths and fish, display conspicuous eye-spots, concentric rings of contrasting colours, that confer survival advantages [12].

Eye-spots can be effective at reducing predation through intimidation or deflection (reviewed in [13]). The intimidation hypothesis proposes that eye-spots, generally those that are large and central, are effective because they frighten the predator. The eye-spots can frighten the predator because they resemble the eyes of vertebrate predators (‘eye mimicry hypothesis’). When predators locate a prey item with eye-spots, the predators may be deterred or startled because the eye-spots resemble the eyes of their own predators, thus giving prey time to escape [14]. The eye-spots can also frighten the predator because they are high-contrast and colourful markings (‘conspicuous signal hypothesis’) [15,16]. Low-level visual features, such as colour, form and luminance [17], exogenously capture attention [18,19]. Eye-spots may therefore be effective because they have low-level features that automatically draw the attention of predators and potentially decrease predation risk. Some evidence supports the conspicuous signal hypothesis [16]. Avian predators are less likely to eat artificial moths with markings that have high contrast, regardless of whether these markings resemble eyes [15,20,21]. However, other studies find no support for the conspicuous signal hypothesis but support the eye mimicry hypothesis. De Bona *et al*. [22] found that natural eye-spots were equally effective compared with real predator eyes at eliciting aversive reactions in great tits. By contrast, other studies find mixed support for both hypotheses [23]. The deflection hypothesis proposes that eye-spots, generally those that are small and marginal, are effective because they attract attention away from the preys' bodies towards the non-vital wings to manipulate where attacking predators direct their strikes, increasing the probability of prey escaping predation. While some studies have found that marginal eye-spots do not affect where predators direct their attacks [24], other studies have found that predators direct their attacks towards marginal eye-spots and away from the insects' bodies [25].

We examined the function of eye-spots in moths and butterflies to evaluate how a potential predator directs its visual attention when encountering prey with eye-spots. Visual search behaviour can be readily investigated with the use of eye-tracking, providing a powerful method for investigating the relationship between prey markings and predator visual attention. While eye-tracking can be performed in non-human animals (e.g. [26,27]), such studies in humans have fewer technical barriers and we therefore used humans as our ‘predators’. Furthermore, there are similarities between humans and other animals in their perceptual systems, such as receptive fields [28], that can result in similar strategies for prey selection. Human subjects have been used as ‘predators’ in previous studies exploring protective coloration [29–32] and have been shown to share some perceptual abilities with other predators [30].

The gaze behaviour of men and women were recorded as they viewed images of butterflies and moths. The images were displayed in their natural form (with the eye-spots intact) or were artificially manipulated (eye-spots were removed, scrambled, or replaced with elliptical or triangular eye-spots with dark or light centres). If eye-spots are effective because they mimic the eyes of predators, we predict predators will direct more attention to the eye-spot regions of moths and butterflies that have their eye-spots intact than to moths and butterflies that have their eye-spots removed or have scrambled/triangular versions of eye-spots. We predict participants to spend similar amounts of time looking at the eye-spot regions of moths and butterflies that had their eye-spots intact and that had elliptical versions of eye-spots with a dark centre because both of these eye-spots resemble eyes. If eye-spots are effective because they are highly salient, we predict predators to direct similar amounts of attention towards the eye-spot regions of moths and butterflies that exhibited eye-spots and any type of modified eye-spot (scrambled, elliptical or triangular) with high contrast (contrast between the eye-spot and the rest of the insect's body).

## 2. Material and methods

### 2.1 Participants

Twenty-three men and 16 women participated in this study at Duke University from November 2012 to March 2013. They were all of European heritage and between the ages of 18 and 30 years old (mean±s.e.: 21.9±0.41 years). We used flyers and e-mails to recruit participants. They earned $15 for their participation.

### 2.2 Butterfly and moth images

**Figure 1.**
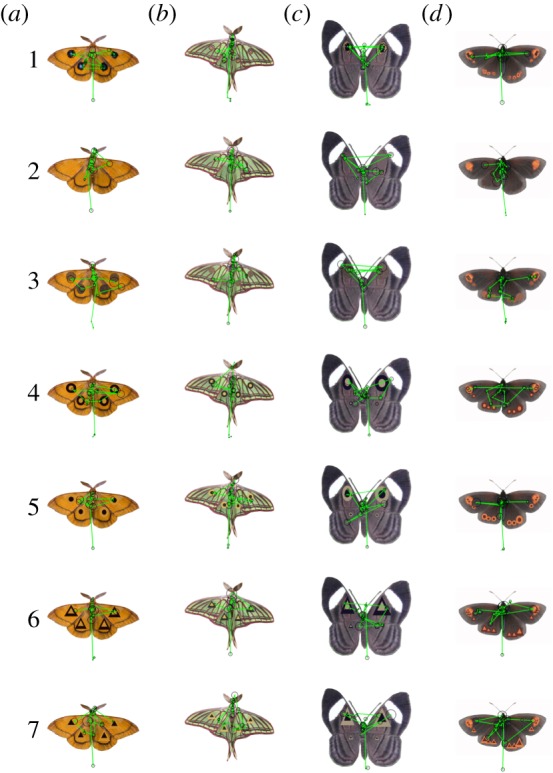
Scanpaths from one female subject looking at images of moths (*a*: *Aglia tau*; *b*: *Graellsia isabellae*) and butterflies (*c*: *Mesosemia asa*; *d*: *Erebia epipsodea*, in the seven different treatments: (1) ‘original’, (2) ‘no eye-spot’, (3) ‘scrambled’, (4) ‘ellipse light,’ (5) ‘ellipse dark,’ (6) ‘triangle light’ and (7) ‘triangle dark.’ The size of the black circles indicates the amount of time the subject was fixating on each location. The subject began looking at the bottom of each image before making a saccade to the moth or butterfly.

We obtained photographs of 70 species of butterflies and moths (table 1) that exhibit eye-spots on their wings from books and online sources. The 70 species belong to seven different families (Carthaeidae (1), Noctuidae (3), Nymphalidae (38), Papilionidae (1), Riodinidae (6), Saturniidae (20) and Sphingidae (1)). The eye-spots were markings on the insect's body parts that were circular and had concentric rings of contrasting colours. We isolated each ‘original’ image of a butterfly or moth and then centred it within a white image (1280×1024 pixels) such that the width of the butterfly or moth extended to the width of the white image (33.72 cm wide; Adobe Photoshop v. 7.0; figure 1*a*). We then created a ‘no eye-spot’ image in which we covered all eye-spots with colours that were immediately surrounding the eye-spots (using the clone stamp tool in Photoshop) so that eye-spots were no longer present (figure 1*b*).

**Table 1.** Species of butterfly or moth used as stimuli in images.

species of butterfly or moth	species of butterfly or moth
*Actias luna*	*Junonia atlites*
*Aglia tau*	*Junonia coenia*
*Anartia jatrophae*	*Junonia evarete*
*Antheraea pernyi*	*Junonia lavinia*
*Antheraea polyphemus*	*Junonia lemonias*
*Apatura iris*	*Junonia orithya*
*Argema mimosae*	*Junonia rhadama*
*Automeris godartii*	*Junonia vestina*
*Automeris io*	*Kallima rumia*
*Automeris larra*	*Lasiommata maera*
*Automeris liberia*	*Lasiommata megera*
*Automeris randa*	*Leucanella apollinairei*
*Bassaris gonerilla*	*Leucanella lynx*
*Bunaea alcinoe*	*Maniola jurtina*
*Caligula japonica*	*Mesosemia asa*
*Carthaea saturnioides*	*Mesosemia hesperina*
*Catacroptera cloanthe*	*Mesosemia telegone*
*Dasypodia selenophora*	*Mesosemia zorea*
*Donuca lanipes*	*Mycalesis terminus*
*Donuca orbigera*	*Opodiphthera eucalypti*
*Enodia anthedon*	*Opodiphthera helena*
*Eochroa trimeni*	*Pararge aegeria*
*Erebia aethiops*	*Parnassius apollo*
*Erebia epipsodea*	*Precis almana*
*Eudia pavonia*	*Precis iphita*
*Eurybia dardus*	*Precis orithya*
*Eurybia lycisca*	*Salamis parhassus*
*Graellsia isabellae*	*Salamis temora*
*Heteronympha merope*	*Saturnia pavonia*
*Heteronympha penelope*	*Smerinthus ocellatus*
*Hypocysta adiante*	*Tisiphone abeona*
*Hypocysta metirius*	*Vanessa gonerilla*
*Inachis io*	*Vindula erota*
*Junonia villida*	*Ypthima asterope*
*Junonia almana*	*Ypthima ceylonica*

Finally, we created five modified versions of each image by overlaying shapes atop the ‘no eye-spot’ image in the exact region where the eye-spots had been located by using custom Matlab (The Mathworks, Natick, MA, USA) scripts. (i) The ‘scrambled’ image was created by randomly repositioning every pixel within ellipses that covered each eye-spot (figure 1*c*). (ii) The ‘ellipse light’ image had two concentric ellipses atop the eye-spot regions, using a dark colour for the outer ellipse and a light colour for the inner ellipse (figure 1*d*). (iii) The ‘ellipse dark’ image was identical to the ‘ellipse light’ image except the dark ellipse was inside the light ellipse (figure 1*e*). (iv) The ‘triangle light’ image was similar to the ‘ellipse light’ image except that triangle shapes were used instead of ellipses (figure 1*f*). (v) The ‘triangle dark’ image was similar to the ‘triangle light’ image except the dark triangle was inside the light triangle (figure 1*g*). For the ‘ellipse’ and ‘triangle’ images, the light and dark colours were chosen for each image as the colours at the 90th and 10th percentile in overall brightness within the image in order to better match the coloration of the insect. The inner shape was 50% the size (in linear dimensions) of the outer shape. For the ‘triangle’ images, the triangles were equal in surface area to the ellipses from the corresponding ‘ellipse’ images and were formed from equilateral triangles stretched to match the aspect ratio of the ellipses.

### 2.3 Experimental procedure

The experimenter (J.L.Y.) told participants that they would be seeing a series of images of butterflies and moths. They were instructed to imagine that they were outside searching for food and specifically looking to find butterflies and moths to eat. They saw seven blocks of images and each block included 70 images. The 70 images within a block included 10 images of each of the seven image versions (‘original’, ‘no eye-spot’, ‘scrambled’, ‘ellipse light’, ‘ellipse dark’, ‘triangle light’ and ‘triangle dark’). Within an image block, a given species of butterfly or moth only appeared one time and was presented in a randomized order. Block order was randomized across participants.

For each image, participants initially saw a white screen with a black dot that was located at the bottom. They used the mouse to click atop the dot so that they were initially fixating on an area where the butterfly or moth stimulus was not present. After clicking the mouse, an image of a butterfly or moth appeared for 3 s. To ensure that participants were actively engaged in the task, after the image disappeared, they had to indicate how likely they would be to select that butterfly or moth as a food source on a scale from 1 (very unlikely) to 10 (very likely). Given that primates, including humans, selectively forage for food [33], we would expect them to exhibit preferences for different food items.

### 2.4 Eye-tracker

We used a Tobii T60 eye-tracker along with Tobii Studio 3.1 and 3.2 (Tobii Technology, Inc., Sweden) to present our images and record the gaze of participants (accuracy: 0.5°; data rate: 60 Hz; binocular tracking). We told participants that we were measuring their pupil size but did not tell them that their eye movements were being monitored until after they finished their trial. The images were displayed using Tobii Studio^^TM^^ software (v. 3.1 or 3.2) on a 1280×1024 pixel monitor (43.18 cm diagonal). Participants were positioned approximately 60 cm from the screen and a chin rest was used to stabilize their heads. The equipment was calibrated before each trial began with nine points. We used the Tobii Velocity-Threshold Identification filter (I-VT filter; gap fill-in: 75 ms; eye selection: average; noise reduction: median; noise reduction samples: 7; velocity calculator window: 20 ms; I-VT classifier threshold: 30° s^−1^; merge adjacent time: 75 ms; merge adjacent angle: 0.5°; discard short fixations: 60 ms) to classify fixations and saccades. This filter classifies eye movements as fixations or saccades based upon the velocity of eye movements; eye movements below and above the velocity threshold (30° s^−1^, in this study) are classified as fixations and saccades, respectively. Eye-tracking data consisted of coordinates of where participants were known to be looking during each sampling point.

### 2.5 Measurements and statistical analysis

Using a customized Matlab program, we drew regions of interest (ROI) around each eye-spot region. In the ‘original’, ‘scrambled’, ‘ellipse light’ and ‘ellipse dark’ images, the ROIs were ellipses that encompassed each eye-spot or modified eye-spot. In the ‘no eye-spot’ image, the ROIs were the same as the ROIs in the ‘original’ image of a given species even though the eye-spots were not visible. In the ‘triangle light’ and ‘triangle dark’ images, the ROIs were triangles that encompassed each modified eye-spot. For each fixation coordinate, we determined which ROI it fell within to determine whether the participant was looking at an eye-spot or modified eye-spot region. We calculated two metrics: the amount of time that elapsed before participants first fixated on the eye-spot or modified eye-spot region and the percentage of time (out of the entire time that the subject was viewing the image) that the subject was fixating an eye-spot or modified eye-spot region.

We calculated Weber contrast using custom Matlab scripts to determine the contrast between the eye-spot regions and the surrounding bodies of the insects. It was calculated as the difference between the mean pixel intensity of the eye-spot region and the mean pixel intensity of the surrounding body divided by the mean pixel intensity of the surrounding body [34]. We measured Weber contrast between each eye-spot and the surrounding body; then we took the mean of the contrasts for each butterfly or moth image. We classified the images as having eye-spots that were darker than the surrounding body (negative Weber contrast) or lighter than the surrounding body (positive Weber contrast); we then calculated the absolute value of the Weber contrast to determine the magnitude of the contrast.

We analysed our data in two steps using SAS (v. 9.3; SAS Institute Inc., Cary, NC, USA). First, we used a generalized linear mixed model (PROC GLIMMIX) to assess whether our independent variables influenced whether or not participants directed their gaze towards eye-spot regions. The independent variables included the treatment (‘original’, ‘no eye-spot’, ‘scrambled’, ‘ellipse light’, ‘ellipse dark’, ‘triangle light’ and ‘triangle dark’), gender of the participant (male or female), the interaction between the treatment and gender of the participant, whether the image was a butterfly or moth, the phylogenetic family of the insect, the total number of eye-spots/modified eye-spots on an image, the mean percentage of surface area each eye-spot/modified eye-spot occupied out of the entire surface area of the insect (‘relative surface area of eye-spots’), the mean distance between the head of the insect and the eye-spots (‘marginality of eye-spots’), contrast type (whether the eye-spots were darker or lighter than the surrounding body), the absolute value of Weber contrast, the interaction between contrast type and the absolute value of Weber contrast, and the edibility rating. Treatment and species name (table 1) were nested within subject identity, which was nested within gender, and were included as a random effects.

Second, we used linear mixed-effects models with repeated measures (PROC MIXED) using only the portion of data in which participants directed gaze towards the eye-spot regions (images in which the participants never looked at any of the eye-spot regions were removed so that underlying model assumptions were met). We examined whether the time viewing eye-spots/modified eye-spots and the latency to fixate an eye-spot/modified eye-spot were influenced by the same independent variables as used in the first step. Treatment and species name (table 1) were nested within subject identity, which was nested within gender, and were included as random effects. We examined all pairwise comparisons among treatments and created contrasts to evaluate these differences. Pairwise comparisons were considered significant if the *p*-value was less than the Bonferonni corrected values.

We also examined the relationship between pupil size (average of the left and right pupil) and the part of the butterfly or moth that the participant was gazing at (body versus eye-spot), treatment, luminance of the body of the butterfly or moth (mean pixel intensity of the body), luminance of the eye-spot regions (mean pixel intensity of the eye-spot region), gender of the participant, interaction between the gender of the participant and treatment, and the edibility rating. The perceived luminance may be slightly different from the pixel luminance because of properties associated with the computer screen [35].

## 3. Results

The number of eye-spots on the butterfly and moth images ranged from two to 24 eye-spots (mean number of eye-spots±s.e.: 5.8±0.59). They covered approximately a tenth of the insect's body (mean±s.e.: 8.8±0.7%; range: 0.03–24.9%). Overall, eye-spots were fixated for approximately a tenth of the viewing time (mean percentage of time looking at eye-spot regions: 6.9±0.12%) but the range was large (0–89.4%). The natural eye-spots on butterflies and moths were usually darker than the surrounding body and had a high contrast (mean Weber contrast±s.e.: −0.12±0.02; range: −0.45–0.26). The elliptical and triangular eye-spots with dark centres had the same contrast (mean Weber contrasts±s.e.: 0.18±0.03) as did the elliptical and triangular eye-spots with light centres (−0.42±0.02). The ‘scrambled’ eye-spots and ‘no eye-spots’ had similar magnitudes of contrast but in opposite directions (‘scrambled:’−0.12±0.02; ‘no eye-spot’: 0.12±0.02)

### 3.1 Probability of looking

**Figure 2.**
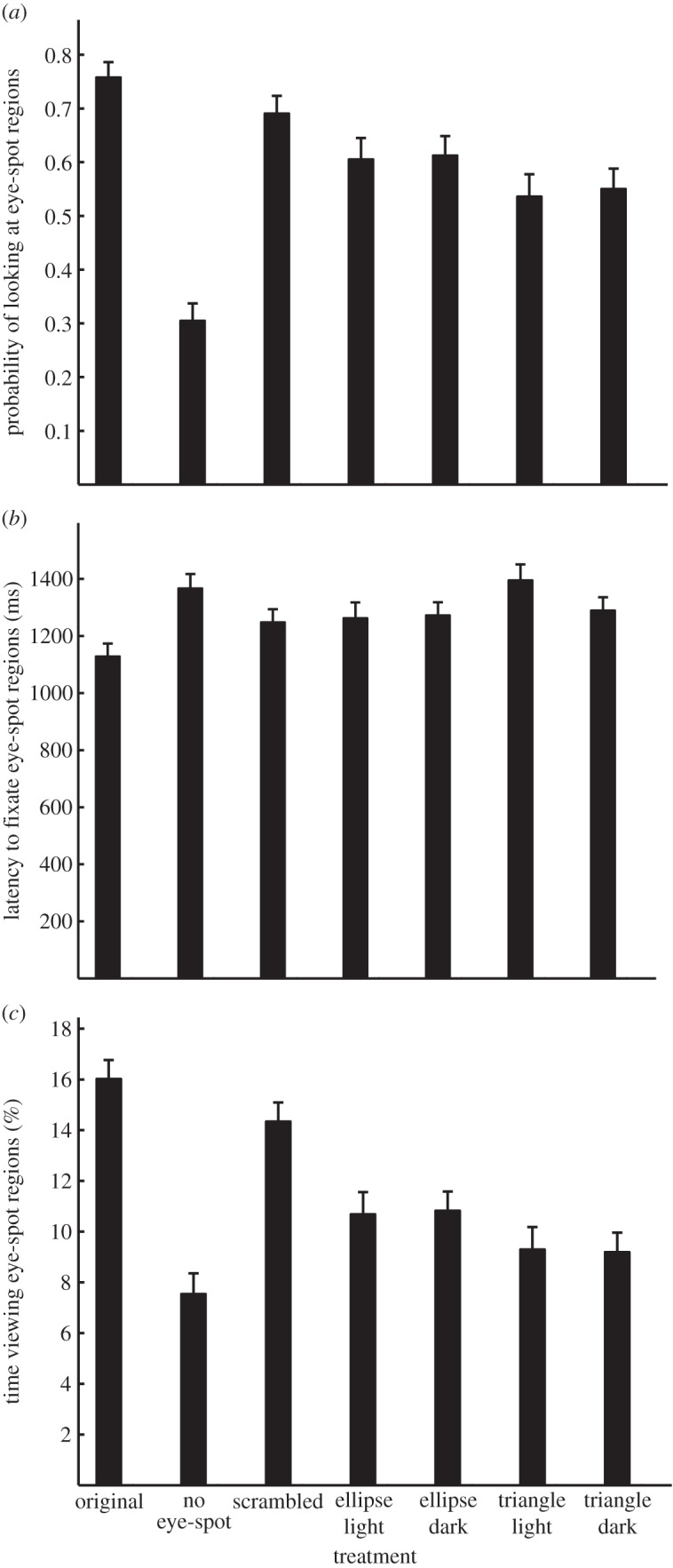
Effect of treatment on gaze behaviour. (*a*) Participants were most likely to gaze at the eye-spot regions in the ‘original’ images compared with all other images. (*b*) They were quickest to fixate the eye-spot region when viewing the ‘original’ images compared with all other images except ‘ellipse dark’. (*c*) They spent more of their time looking at the eye-spot regions in the ‘original’ images compared with all other images (with the exception of time viewing the ‘scrambled’ image). LSMeans and standard-error values are shown.

The type of treatment (‘original’, ‘no eye-spot’, ‘scrambled’, ‘ellipse light’, ‘ellipse dark’, ‘triangle light’ and ‘triangle dark’) influenced whether participants looked at eye-spot regions or not (figure 2*a* and table 2). Participants were more likely to look at the eye-spot regions in moths compared with butterflies. They were also more likely to look at the eye-spot regions when there were more eye-spots, the mean surface area of the eye-spots was greater, and the eye-spots were closer to the head of the insect. Participants were more likely to look at eye-spot regions that were darker than the surrounding body but the magnitude of contrast did not affect whether participants looked at eye-spot regions or not. Participants indicated that they were more likely to select the butterfly or moth as a food source when they looked at the eye-spot regions.

**Table 2.** The effects of variables on whether the participants looked at the eye-spot regions or not (‘probability look’), the amount of time participants spent looking at the eye-spot regions (‘time looking’), and their latency to fixate the eye-spot regions (‘latency to fixate’). Asterisks indicate whether the comparison is significant.

	*F*_num d.f., den d.f._(*p*)		
variable	probability look	latency to fixate	time looking
treatment	69.80_6,264.6_ (<0.0001)*	9.00_6,246_ (<0.0001)*	38.81_6,276_ (<0.0001)*
gender	0.33_1,36.46_ (0.57)	0.02_1,36.7_ (0.90)	0.07_1,36.5_ (0.80)
treatment×gender	0.67_6,203.8_ (0.68)	0.31_6,188_ (0.93)	0.81_6,217_ (0.56)
insect type	5.79_1,2242_ (0.016)*	20.21_1,2175_ (<0.0001)*	0.24_1,2270_ (0.62)
phylogenetic family	5.83_6,2548_ (<0.0001)*	4.95_6,2113_ (<0.0001)*	6.70_6,2203_ (<0.0001)*
number of eye-spots	178.94_1,2774_ (<0.0001)*	13.04_1,2155_ (0.0003)*	91.88_1,2241_ (<0.0001)*
marginality of eye-spots	94.95_1,2888_ (<0.0001)*	41.83_1,2063_ (<0.0001)*	108.09_1,2141_ (<0.0001)*
relative surface area of eye-spots	81.81_1,3218_ (<0.0001)*	60.30_1,1830_ (<0.0001)*	214.27_1,1888_ (<0.0001)*
contrast type	8.85_1,12049_ (0.0029)*	0.03_1,6519_ (0.87)	1.80_1,6558_ (0.18)
Weber contrast (absolute value)	0.81_1,12049_ (0.37)	0.21_1,6577_ (0.65)	0.00_1,6560_ (0.99)
contrast type×Weber contrast (absolute value)	3.72_1,12049_ (0.054)	1.40_1,5629_ (0.24)	2.64_1,5467_ (0.10)
edibility rating	14.39_1,10822_ (0.0001)*	0.28_1,5341_ (0.60)	10.92_1,5416_ (0.001)*

Participants were more likely to look at the eye-spot regions in images that exhibited natural eye-spots compared with images with no eye-spots or modified eye-spots (‘scrambled’, ‘ellipse light’, ‘ellipse dark’, ‘triangle light’ and ‘triangle dark’; table 3 and figure 2*a*). They were statistically less likely to look at eye-spot regions in the images lacking eye-spots compared with all other images. The scrambled eye-spots were more likely to draw attention than the elliptical or triangular eye-spots. Participants were not more likely to look at the elliptical and triangular eye-spots with dark centres compared with light centres.

**Table 3.** Pairwise comparisons across treatment groups for whether the participants looked at the eye-spot regions or not (‘probability look’), the amount of time participants spent looking at the eye-spot regions (‘time looking’), and their latency to fixate the eye-spot regions (‘latency to fixate’). Asterisks indicate whether the comparison is significant using a Bonferroni correction (*p*-value=0.05/21 comparisons=0.002).

	*F*_num d.f., den d.f._ (*p*)		
treatment comparison	probability look	latency to fixate	time looking
original versus			
no eye-spot	18.15_1,291.7_ (<0.0001)*	26.84_1,321_ (<0.0001)*	132.9_1,331_ (<0.0001)*
scrambled	3.31_1,224.5_ (0.0011)*	10.71_1,141_ (0.0013)*	7.55_1,171_ (0.0066)
ellipse light	6.19_1,370.3_ (<0.0001)*	8.97_1,304_ (0.003)	55.51_1,312_ (<0.0001)*
ellipse dark	6.25_1,301.3_ (<0.0001)*	11.8_1,236_ (0.0007)*	58.45_1,254_ (<0.0001)*
triangle light	8.66_1,369.7_ (<0.0001)*	33.96_1,336_ (<0.0001)*	84.95_1,338_ (<0.0001)*
triangle dark	8.61_1,300.1_ (<0.0001)*	14.1_1,245_ (0.0002)*	97.26_1,265_ (<0.0001)*
no eye-spot versus			
scrambled	15.34_1,273.4_ (<0.0001)*	6.46_1,342_ (0.012)	83.36_1,349_ (<0.0001)*
ellipse light	9.66_1,608.8_ (<0.0001)*	3.25_1,752_ (0.072)	12.46_1,675_ (0.0004)*
ellipse dark	12.98_1,208.3_ (<0.0001)*	4.45_1,284_ (0.036)	20.79_1,302_ (<0.0001)*
triangle light	7.48_1,604.6_ (<0.0001)*	0.23_1,800_ (0.63)	3.8_1,712_ (0.052)
triangle dark	10.39_1,207_ (<0.0001)*	2.88_1,298_ (0.091)	5.06_1,316_ (0.025)
scrambled versus			
ellipse light	3.31_1,354.9_ (0.001)*	0.10_1,321_ (0.75)	25.69_1,324_ (<0.0001)*
ellipse dark	3.21_1,286.9_ (0.0015)*	0.33_1,255_ (0.57)	25.95_1,271_ (<0.0001)*
triangle light	5.81_1,352.6_ (<0.0001)*	10.14_1,352_ (0.0016)*	47.2_1,350_ (<0.0001)*
triangle dark	5.62_1,283.6_ (<0.0001)*	0.89_1,263_ (0.35)	53.76_1,280_ (<0.0001)*
ellipse light versus			
ellipse dark	0.23_1,654.4_ (0.82)	0.03_1,667_ (0.86)	0.03_1,598_ (0.87)
triangle light	2.91_1,193.3_ (0.004)	11.07_1,194_ (0.001)*	4.49_1,220_ (0.035)
triangle dark	1.71_1,648.7_ (0.087)	0.23_1,666_ (0.63)	2.99_1,602_ (0.084)
ellipse dark versus			
triangle light	2.37_1,652.9_ (0.018)	4.76_1,715_ (0.030)	3.12_1,634_ (0.078)
triangle dark	2.65_1,191_ (0.0086)	0.18_1,180_ (0.67)	6.24_1,209_ (0.013)
triangle light versus			
triangle dark	0.43_1,645.5_ (0.67)	3.5_1,713_ (0.062)	0.01_1,638_ (0.90)

### 3.2 Latency to fixate

The latency to initially fixate an eye-spot region depended on the treatment (table 2 and figure 2*b*). Participants were quicker to look at the eye-spot regions of moths than those of butterflies. They were faster to fixate eye-spots when there were more eye-spots, the mean surface area of the eye-spots was greater, and the eye-spots were closer to the head of the insect. The contrast of the eye-spots did not influence latency to fixate the eye-spot regions. The latency to fixate eye-spot regions was unrelated to participants' indication of whether they would select the butterfly or moth as a food source.

Participants were faster to detect the eye-spot regions in the images that exhibited natural eye-spots compared with images with no eye-spots or modified eye-spots (with the exception of ellipse ‘ellipse light’; table 3 and figure 2*b*). The eye-spot regions in the ‘scrambled’ images were also more quickly detected than those in the ‘triangle light’ images. Similar amounts of time were spent looking at elliptical and triangular eye-spots regardless of whether the inside of the respective shapes was light or dark coloured, with the exception of ‘ellipse light’ and ‘triangle light’.

### 3.3 Time looking

The amount of time that participants fixated eye-spot regions varied depending on the treatment (table 2 and figure 2*c*). They directed more attention towards the eye-spot regions when there were more eye-spots, the mean surface area of the eye-spots was greater, and the eye-spots were closer to the head of the insect. Neither the type of contrast nor the magnitude of contrast was a significant predictor of the amount of time participants fixated eye-spots. Participants indicated that they were more likely to select the butterfly or moth as a food source when they spent more time looking at the eye-spot regions.

Participants spent more time looking at the eye-spot regions in the images that exhibited natural eye-spots compared with images with no eye-spots or modified eye-spots (with the exception of scrambled eye-spots). They spent less time looking at eye-spot regions in images that lacked eye-spots compared with ‘scrambled’, ‘ellipse dark’ and ‘ellipse light’ images. They directed more attention towards ‘scrambled’ eye-spots than the other modified eye-spots.

### 3.4 Pupil size

Pupil size was related to treatment (*F*_6,256_=5.12, *p*<0.0001), luminance of the body of the butterfly or moth (*F*_1,4829_=454.9, *p*<0.0001) and the part of the butterfly or moth that the participant was gazing at (*F*_1,17000_=13.42, *p*=0.0003), but unrelated to the gender of the participant (*F*_1,37_=0.50, *p*=0.49), the interaction between the gender of the participant and treatment (*F*_6,211_=1.04, *p*=0.40), the luminance of the eye-spot regions (*F*_1,10000_=3.35, *p*=0.067) and the edibility rating (*F*_1,11000_=1.11, *p*=0.29). Pupil size was larger when participants were gazing at the eye-spot regions compared with the surrounding body (LSMean±s.e.: eye-spots: 2.68±0.046 mm; body: 2.67±0.046 mm) but was smaller when the luminance of the body was bright.

## 4. Discussion

Even though the eye-spot regions only occupied a fraction of the butterfly or moths' bodies, they still attracted large amounts of attention. Humans were more likely to look at the natural eye-spots of butterflies and moths than the eye-spots of butterflies and moths that had modified eye-spots (scrambled, elliptical or triangular). They were faster to initially fixate the eye-spots of butterflies and moths with their natural eye-spots compared with butterflies and moths that had modified versions of eye-spots, except for the elliptical eye-spots with light centres. They also spent the most time looking at eye-spots of butterflies and moths with their natural eye-spots compared with butterflies and moths that had modified versions of eye-spots (with the exception of the scrambled eye-spots). They were not simply looking at eye-spots because they were located in salient positions on the insects. When the eye-spots were removed, participants had a lower probability of looking at the eye-spot regions compared with when the natural eye-spots were still intact. Interestingly, participants' pupil sizes were larger when they viewed the eye-spot regions compared with the rest of the butterfly or moth body, indicating a greater arousal level (potentially due to intimidation) when viewing eye-spots.

We found mixed support for the conspicuous signal hypothesis, which posits that eye-spots are effective at deterring predators through intimidation because they are highly salient signals [15,16,20]. The natural eye-spots of the butterflies and moths did not have the highest contrast levels compared with the other modified eye-spots. Despite this, they still attracted the most attention and humans were generally quickest to fixate them compared with the eye-spot regions of butterflies and moths lacking eye-spots or exhibiting modified eye-spots. Furthermore, eye-spots with scrambled pixels had the same contrast as natural eye-spots but humans directed less attention towards the scrambled eye-spots compared with the natural eye-spots. Interestingly, the natural eye-spots of butterflies and moths were usually darker than the surrounding body of the insect and had a high contrast (large and negative Weber contrast). Previous research in humans shows that luminance decrements (i.e. negative Weber contrast) are more visually salient than luminance increments (i.e. positive Weber contrast) [36,37], and contrast may therefore be contributing to eye-spot salience but not driving its effect.

The number, size and marginality of eye-spots affected gaze behaviours. Humans were more likely to look at eye-spot regions, be faster to look at eye-spot regions and spend more time looking at eye-spot regions when the butterfly or moth had more eye-spots and those eye-spots were larger. Consistent with this gaze behaviour, Stevens *et al.* [20] found that artificial prey exhibiting more eye-spots and larger eye-spots were more likely to survive than prey exhibiting eye-spots with opposite properties. This suggests that predators' visual attention towards numerous and large eye-spots impacts predator hunting success. Numerous and large eye-spots may overload the sensory system of predators, supporting the conspicuous signal hypothesis [16]. In addition, participants spent more time looking at eye-spot regions and were faster to first fixate eye-spot regions when the eye-spots were located closest to the heads of the insects, suggesting that marginal eye-spots are not the most effective at drawing predator attention and may therefore have limited effects on deflecting attack [24].

We found minimal support for the eye mimicry hypothesis, which states that eye-spots are effective at reducing predation through intimidation because they resemble the eyes of real predators [14]. Participants had a higher probability of looking at natural eye-spots and spent more time looking at natural eye-spots compared with elliptical eye-spots (with dark centres) that also resembled eyes. In addition, participants spent similar amounts of time looking at and initially fixating elliptical eye-spots regardless of whether the inner ellipse was dark (and thus more closely mimicked a real eye) or light. Eye-spots with scrambled pixels, which did not resemble eyes except in their elliptical shape, attracted more attention than elliptical eye-spots with dark centres. Furthermore, elliptical eye-spots with dark centres did not have higher probabilities of being fixated and were not fixated for longer amounts of time than triangular eye-spots with dark centres, the latter of which did not resemble eyes in shape but exhibited similar contrast levels to the elliptical eye-spots. Therefore, the resemblance of eye-spots to real eyes seems unlikely to be the driving factor for attracting or maintaining attention because more eye-like stimuli (elliptical eye-spots with dark centres) did not attract more attention than less eye-like stimuli (elliptical eye-spots with light centres, triangular eye-spots and scrambled eye-spots; albeit natural eye-spots did attract more attention than any of these modified eye-spots [15]). Similarly, avian predators were deterred by eye-spots regardless of whether the eye-spots were more or less eye-like [38]. An experiment probing the cognitive biases of starlings also failed to find support for the eye mimicry hypothesis: starlings did not show an increased aversion to eye-spots after being exposed to alarm calls, suggesting that they did not categorize the eye-spots as a threat [39]. Future experiments using modified eye-spots that more closely resemble the eyes of real predators (rather than using simplified ellipses; see also [20]) would be informative to further probe this hypothesis.

The visual systems of predators can significantly impact their abilities to detect and capture prey. Predators may be unable to see certain wavelengths that prey use when signalling with each other [40,41] and this may decrease the predators' abilities to detect that prey. Predators may also fail to note prey that mimic environmental features [42] or blend in with their environments [43]. Alternatively, predator attention may be drawn to conspicuous prey markings that can lead to predator startle responses [44]. Rather than attacking the vital body region of prey, predators may even mistakenly target the conspicuous wing markings of prey and allow the prey to successfully escape. Our results demonstrate that the visual system of human predators can also be influenced by the appearance of prey. In particular, human attention was drawn towards eye-spot markings on butterflies and moths and this attention could affect their abilities to successfully capture prey in natural environments.

## Data accessibility

The data supporting this article are available in the Dryad Digital Repository: http://dx.doi.org/10.5061/dryad.r8dv2.
